# 3D Printed Hydrogel Microneedle Arrays for Interstitial Fluid Biomarker Extraction and Colorimetric Detection

**DOI:** 10.3390/polym15061389

**Published:** 2023-03-10

**Authors:** Mahmood Razzaghi, Amir Seyfoori, Erik Pagan, Esfandyar Askari, Alireza Hassani Najafabadi, Mohsen Akbari

**Affiliations:** 1 Laboratory for Innovations in Microengineering (LiME), Department of Mechanical Engineering, University of Victoria, Victoria, BC V8P 5C2, Canada; 2 Terasaki Institute for Biomedical Innovations, Los Angeles, CA 90050, USA; 3 Biotechnology Center, Silesian University of Technology, Akademicka 2A, 44-100 Gliwice, Poland

**Keywords:** microneedle, microneedle array, 3D printing, hydrogel, interstitial fluid, biomarker, detection

## Abstract

To treat and manage chronic diseases, it is necessary to continuously monitor relevant biomarkers and modify treatment as the disease state changes. Compared to other bodily fluids, interstitial skin fluid (ISF) is a good choice for identifying biomarkers because it has a molecular composition most similar to blood plasma. Herein, a microneedle array (MNA) is presented to extract ISF painlessly and bloodlessly. The MNA is made of crosslinked poly(ethylene glycol) diacrylate (PEGDA), and an optimal balance of mechanical properties and absorption capability is suggested. Besides, the effect of needles’ cross-section shape on skin penetration is studied. The MNA is integrated with a multiplexed sensor that provides a color change in a biomarker concentration-dependent manner based on the relevant reactions for colorimetric detection of pH and glucose biomarkers. The developed device enables diagnosis by visual inspection or quantitative red, green, and blue (RGB) analysis. The outcomes of this study show that MNA can successfully identify biomarkers in interstitial skin fluid in a matter of minutes. The home-based long-term monitoring and management of metabolic diseases will benefit from such practical and self-administrable biomarker detection.

## 1. Introduction

Continuous monitoring of relevant biomarkers is required to treat and manage chronic diseases to adjust treatment as the disease state changes [1,2,3]. Conventional methods for detecting biomarkers are based on invasive blood sampling and relevant laboratory testing [4]. Sensors that can detect biomarkers in urine, tears, sweat, and saliva have been developed to solve the invasiveness of blood sampling [5,6,7]. Nevertheless, the concentration and dynamics of the biomarkers available in the mentioned bodily fluids are limited and poorly correlated with their existence in blood, making them typically of low diagnostic relevance [8,9]. Transdermal biosensors detect biomarkers by analyzing interstitial fluid (ISF) found in the dermis’ lowermost skin layer [10,11,12]. Interstitial fluid has the most comparable molecular composition to blood plasma compared to other bodily fluids [13], in addition to having other unique characteristics such as biomarkers of medical relevance [10,14]. Dermal tissue interstitial fluid generated by blood transcapillary filtration is an appealing biomarker source because about 80% of its components are shared with plasma, and it contains specific unique biomarkers particular to certain disorders like melanoma [15,16]. In addition, some biomarkers available in ISF are not available in blood plasma [17]. Transdermal biosensing allows for the non-invasive or minimally invasive continuous monitoring of patient health status, which can move us closer to personalized and precision medicine [10,18,19]. 

There are limited techniques for ISF sampling, including suction blisters, microdialysis, open-flow microperfusion, reverse iontophoresis, and microneedle patches [14,20]. However, procedures like suction blisters are usually complicated, often cause patient discomfort, and hold a potential risk of infection. Recently, microneedle arrays (MNAs) have shown great potential and some success in extracting skin ISF [12]. Extraction and analysis of ISF using microneedle array devices allows for minimally invasive patient health monitoring in point-of-care settings. Straightforward and efficient methods for a complete analysis of interstitial fluid can result in game-changing advancements in diagnostic practices. These methods are not only painless and minimally invasive, but they are also appropriate for point-of-care and resource-constrained settings [18]. However, due to the stratum corneum (SC) barrier that is the skin’s outer layer, the ISF metabolites cannot easily permeate the skin surface for testing [21]. As a result, the suction blister technique is employed to remove ISF for analysis, which can only be performed by qualified experts in clinics and can cause discomfort or even infection [14]. Microneedles (MNs) that can penetrate the SC with little invasiveness and extract ISF by capillary force or vacuum suction have been developed [22,23,24,25]. Microneedle-based techniques have been presented as efficient methods for basic ISF extraction with the potential for diagnostic integration [26,27,28,29]. 

Various types of MNAs use different ways to extract skin interstitial fluid. Hollow MNs work based on negative pressure [26,30]; capillary force is used by porous MNs [26,30]; and the most recent, hydrogel-based MNAs use material absorption [11,26]. Hydrogel-based MNAs with shorter lengths and sharper tips than hypodermic needles allow for efficient penetration of the skin SC and the development of microscale interstitial fluid extraction channels [18]. In comparison to other MNAs, hydrogel-based MNAs have several advantages, such as improved and faster ISF extraction, excellent biocompatibility, cheaper fabrication costs, higher production output, and, most importantly, the convenience of insertion and removal without harming the skin [12,26,31,32,33]. The attachment of biosensors on MNs allows for insitu characterization of interstitial fluid [11,12]. Swellable polymeric MNs have also been shown to be effective for ISF absorption [34]. Although microneedle arrays are simple to apply, the current methods necessitate a lengthy sampling period as well as additional equipment for operation and analysis [35]. MNAs with improved ability to swell were demonstrated [10,12,33], in which centrifugation was utilized to extract the adsorbed ISF, and laboratory apparatuses were required for offline analysis. A new study used antibody-coated polystyrene MNAs to capture protein biomarkers in ISF for offline enzyme-linked immunosorbent assays with significantly increased sensitivity [36]. However, the functionalization of MNs and the requirement of a microplate reader for fluoroimmunoassay complicates the process. Recently, efforts have been undertaken to insert enzymes and dye molecules inside MNs for colorimetric detection of hyperglycemia for point-of-care diagnosis [37]. On the other hand, the leak of sensing reagents and by-products created by the enzymatic reactions of MNAs into the skin is a big concern. [38,39]. Although it has been demonstrated that conjugating glucose oxidase on nanoparticles in MNA-based colorimetric glucose sensors prevents the enzyme from escaping into skin tissue, leakage of dye molecules and H_2_O_2_ is not avoided, adding complications to MNA manufacture. [40]. Nevertheless, most MNA-based diagnostic techniques are either complex fabrication processes or ISF post-processing [18]. Recent research has focused on hydrogel-forming MNs with the added capability of real-time electrically tracking health conditions. The conductive hydrogel microneedle array electrode was demonstrated, allowing for on-needle pH sensing without the requirement for post-processing. The MNA fabrication involved using swellable dopamine-conjugated hyaluronic acid hydrogel embedded with poly(3,4-ethylene-dioxythiophene): polystyrene sulfonate (PEDOT: PSS) to improve conductivity. The catechol-quinone chemistry intrinsic to dopamine was utilized to measure pH in interstitial fluid. The impact of PEDOT: PSS on the mechanical strength and swelling capacity of the MNA array was investigated [41]. An on-needle sensing mechanism has some risks, like health risks due to direct contact of required sensing reagents with skin tissue.

Improvements in printing resolution, feature precision, and the availability of new printing materials have enabled three-dimensional (3D) printing to fabricate different types of MNs [42,43,44,45]. Three-dimensional printing techniques can fabricate more sophisticated and complicated MN structures than conventional methods [46]. MNs with various structures can be made using 3D printing technology in a single step, and the 3D printer’s high resolution ensures that the arrays can be formed in detail [47]. 

**Figure 1:**
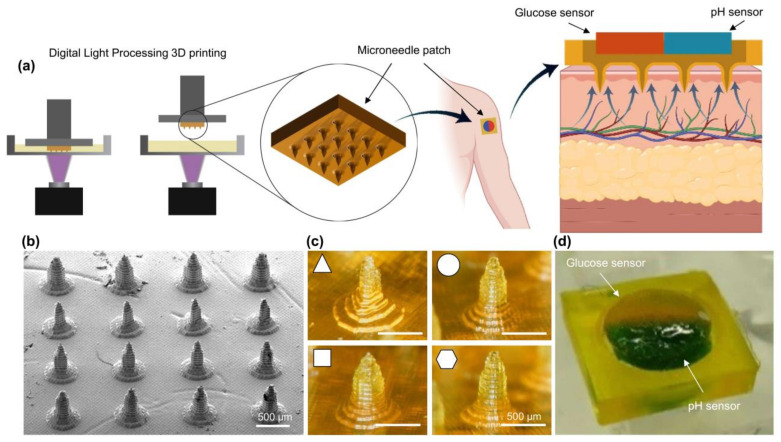
Schematic illustration of the application process of (**a**) Hydrogel-based microneedle patch; (**b**) Scanning electron microscopy (SEM) image of MNA; (**c**) Printed microneedles with different cross-sections; (**d**) Microneedle patch.

This article presents 3D-printed colorimetric MNAs for multiplexed transdermal metabolite detection to solve the abovementioned problems. MNs are composed of crosslinked poly(ethylene glycol) diacrylate (PEGDA), which is swellable with good mechanical strength. PEGDA is a biocompatible hydrogel [48]. The applied sensor is detachable, can sense metabolites in situ, in real-time colorimetrically, and report the pH and concentrations of glucose in ISF. This study aims to investigate the feasibility of using PEGDA hydrogel MNAs for ISF detection applications and to find optimum specifications. In this regard, the fabrication of MNAs using the 3D printing process is assessed, and then the capability of MNAs with different specifications for liquid extraction and skin penetration, and colorimetric sensing is evaluated. Based on the outcomes, the MNA with the best specifications is suggested. By providing self-administrable continuous monitoring of biomarkers in ISF, which is suitable for efficient home-based use with high patient compliance, this technique offers prospects for safe and long-term monitoring and optimal management of chronic diseases. The fabrication and application process of hydrogel-based microneedle arrays are depicted in Figure 1. 

## 2. Results and Discussion

### 2.1. Fabrication of MNAs Using 3D Printing

**Figure 2:**
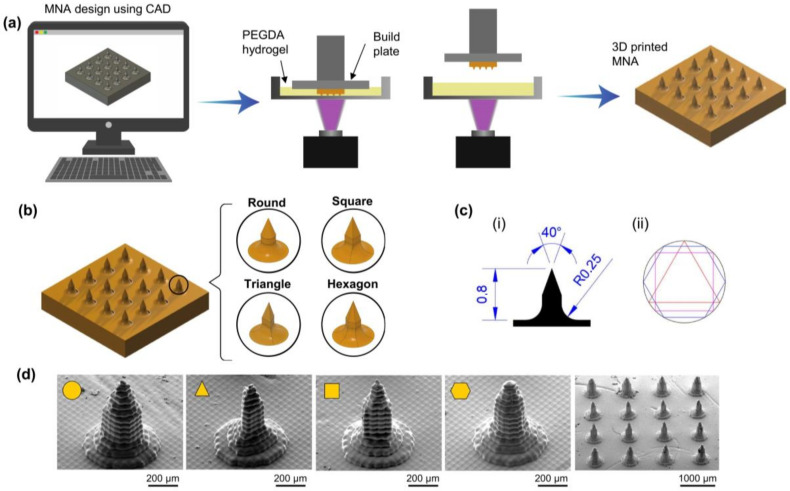
Fabrication process of microneedle arrays. (**a**) Schematic illustration of DLP printing of MNAs; (**b**) CAD designs of MNAs with different cross-section shapes; (**c**) Dimensions of needles, (i) height, tip angle, and corner radius of the needles, (ii) comparison of needle sections with different shapes; (**d**) SEM images of the printed microneedles with different cross-section shapes.

MNAs were fabricated using the digital light processing (DLP) bioprinting method. The process of bioprinting is shown schematically in Figure 2a. Computer-aided design (CAD) software was used to generate the MN array’s (four by four) 3D model. Digitally, the 3D model was sliced into several cross-sectional images. Every digital image was sent to a digital micromirror device (DMD), which produced patterned light (405 nm), which was then sent through a projection lens and concentrated on the surface of the photocurable precursor solution. The projection light transformed the liquid photocurable precursor solution into a solid patterned layer. To create a 3D MNA, this process was repeated layer by layer. In this study, we used PEGDA with a molecular weight of 700 Da (PEGDA 700) as a monomer, tartrazine as a photo absorber, and lithium phenyl-2,4,6-trimethylbenzoylphosphinate (LAP) as a photoinitiator.

The MNAs with round, triangle, square, and hexagon cross-section shapes were designed and fabricated to investigate the properties and characterization. MNAs were designed to have a length of 800 µm and a cone angle at the tip of 40°, as shown in Figure 2c. The SEM images (Figure 2d) showed that the MNAs with different cross-section shapes exhibit appropriate structure. MNAs with different concentrations of PEGDA werealso 3D printed, ranging from 15% to 85%.

**Figure 3:**
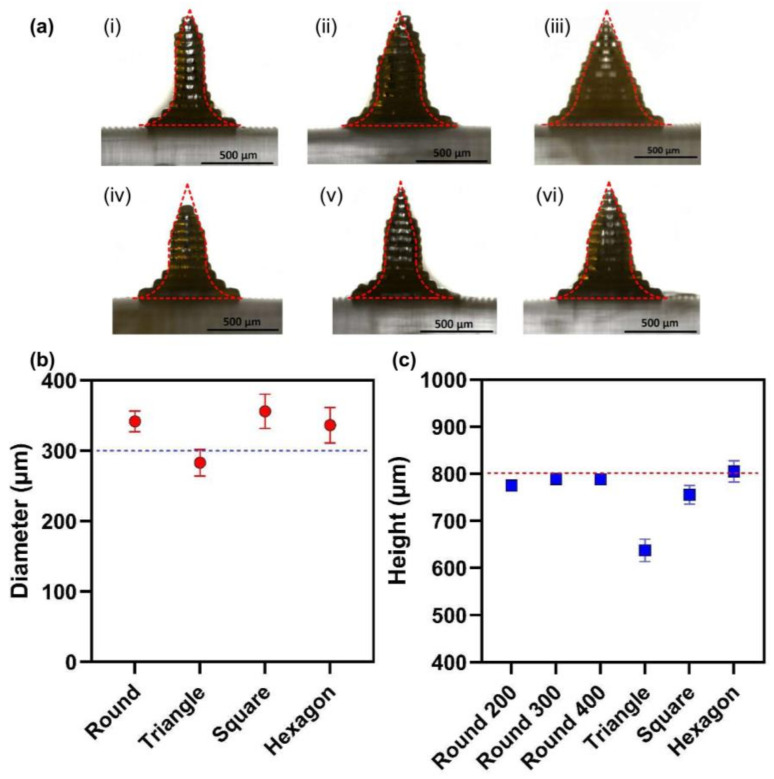
(**a**) A graphical size comparison between designed and printed MNs, (i) round MN with 200 µm diameter, (ii) round MN with 300 µm diameter, (iii) round MN with 400 µm diameter, (iv) triangle section MN with 300 µm, (v) square section MN with 300 µm, (vi) hexagon section MN with 300 µm, diameter; (**b**) Chart comparing the diameter of designed MNs with the nominal diameter; (**c**) Chart comparing the height of designed MNs with the nominal height.

To compare the designed MNAs and printed ones dimensionally, MNAs with different cross-section shapes, with 800 µm height and 300 µm (surrounded circle) diameter, and round section MNAs with 200, 300, and 400 µm diameters were 3D printed. Figure 3 shows the results of the measurements of the MNA. The results showed that the printed MNAs have good dimensional precision. 

### 2.2. Swelling Properties

**Figure 4:**
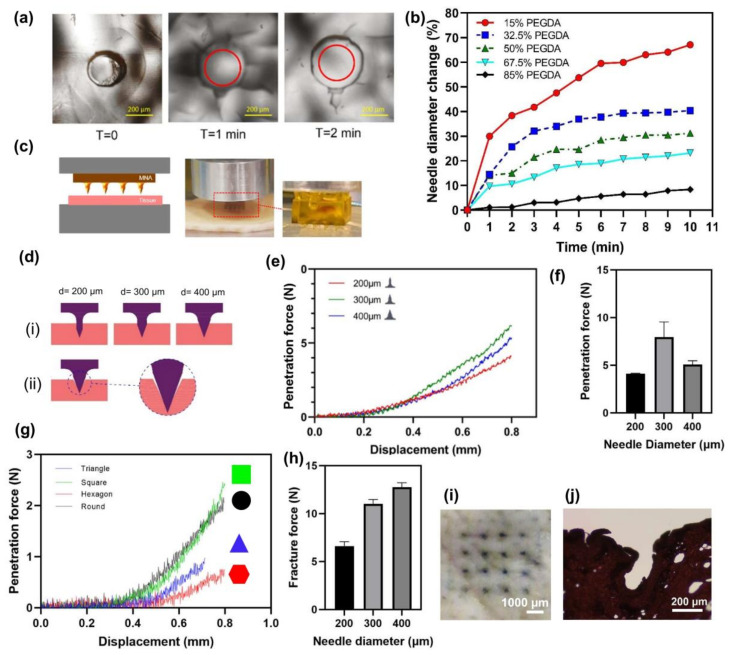
Swelling and mechanical properties. (**a**,**b**) Changes of the MN diameter at different times of swelling test; (**c**) Mechanical testing device; (**d**) Schematic illustration of MNs penetration with different diameters; (**e**) Penetration force versus displacement for round MNAs with different diameters; (**f**) Maximum penetration force of round MNAs with different diameters; (**g**) Penetration force for MNAs with different cross-section shape; (**h**) Fracture force of MNAs with different needle diameters; (**i**) Top view of pig skin after MNA insertion using blue dye; and (**j**) Stained section of pig skin penetrated by the MNA.

Non-dissolvable hydrogel-based MNAs could be used for the rapid and significant absorption of extracted ISF for colorimetric analysis if they were highly swellable. To study the swelling properties, MNAs with different concentrations of PEGDA were 3D printed, ranging from 15% to 85%. Table 1 shows the compositions of different formulations of MNAs. Phosphate-buffered saline (PBS) solution was used to investigate the ability of MNAs to extract ISF. MNAs with varying concentrations of PEGDA were placed in PBS solution for 10 min, and the increase of the diameter of needles was measured as an index for liquid absorption and swelling. All five different 3D-printed microneedles were immersed in PBS to determine the change in the diameter of needles due to swelling over time. A more significant change in diameter could be an index for the capability of higher wicking properties. A faster weight change indicates a more rapid liquid absorption in the microneedle array. Figure 4a shows the needle diameter change in a 15% PEGDA microneedle at the start and after one and two min of immersion. Figure 4b also depicts the changes in diameter in 10 min of immersion for all samples. As the MNAs have high swellability, they become saturated in a few minutes after soaking in PBS. 

The swelling property of MNAs is related to their material. Decreasing the concentration of PEGDA in MNAs results in lower density and provides more space to absorb more water. As can be seen in Figure 4b, the MNA with a 15% PEGDA concentration, has about a 67.1% increase in its needle diameter which shows its high swellability, while for the MNA with an 85% PEGDA concentration, the needle diameter increase in 10 min is about 8.4%. 

### 2.3. Mechanical Properties

Another property required for using MNAs as a colorimetric detection device is good mechanical properties strong enough to penetrate the skin. To evaluate the mechanical properties of MNAs, a compression mechanical testing system was used (Figure 4c), in which the required force for the penetration of MNAs in pig skin was measured. The MNAs fabricated with PEGDA concentrations less than 50% (15% and 32.5%) failed in the penetration experiment. The effect of needle diameter on the penetration force was assessed using mechanical testing MNAs with 200 µm, 300 µm, and 400 µm needle diameters. The results showed that the MNA with a needle diameter of 300 µm has a higher penetration force than MNAs with 200 µm and 400 µm needle diameters. It is evident that the penetration force is a function of the area that contacts the tissue during the penetration. It is expected that with increasing the needle diameter, the penetration force is increased. The MNA with a 300 µm needle diameter has a higher penetration force than the 400 µm. Figure 4d shows the penetration of MNAs with different needle diameters schematically. As can be seen in Figure 4d(ii), in the tip area, the sharp edge can decrease the contact area of the needle with the tissue. This could be a reason for a lower penetration force of 400 µm needle diameter MNA compared to the one with 300 µm as it has a higher tip angle area and lowers the remaining needle external needle area. 

The effect of needle cross-section shape on penetration force was studied. The MNAs with a diameter of 300 µm and round, hexagon, square, and triangle cross-section shapes were used for this study. The penetration force seems to be a function of two parameters: needle contact area and the number of cross-section shape vertices. The penetration is increased with increasing the contact area of the needle and the tissue. Furthermore, the penetration force is decreased with increasing the number of cross-section shape vertices [49]. Table 2 shows the value of the contact perimeter (as an index of contact area) and the number of vertices for MNAs with different cross-section shapes. Based on the results, a hexagon cross-section shape with a 200 µm needle diameter has a lower penetration force as an advantage. 

Figure 4h depicts the fracture force of 85% PEGDA concentration MNAs with different needle diameters. The measured fracture forces were between about 6.3 to 13.1 N and the results showed that the fracture forces of MNAs are higher than their relevant penetration forces, indicating that they are safe for penetrating the skin. Other research also reported a fracture force in the range of about 2 to 10 N for their 3D-printed MNAs [50]. Figure 4i,j also show the top view of pig skin after MNA insertion using blue dye, and the stained section of pig skin penetrated by the MNA, respectively. 

### 2.4. Sensing

**Figure 5:**
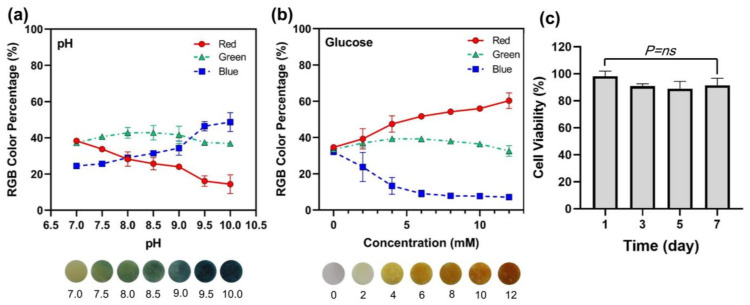
(**a**) RGB color percentage changes for the pH sensing; (**b**) RGB color percentage changes for the glucose sensing; (**c**) Cell viability test results for 50% PEGDA MNA.

The sensing properties of MNAs were evaluated using two biomarkers, pH and glucose. Physiological pH monitoring is extremely important in clinical practice because it provides information about the body’s regulatory systems [51]. A slight change in physiological pH can have repercussions on cellular, tissue, and organ functions and can thus be used to detect disease early [51]. Monitoring the pH of both tissues and organs is critical in the diagnosis of many diseases [41]. In this study, pH levels ranging from 7.0 to 10.0 were detected colorimetrically by MNAs. The representative images of the color change of the MNA sensor in the detection of different pH values and the relevant changes in the red, green, blue (RGB) percent (blue) are depicted in Figure 5a. 

Normal fasting blood glucose levels range from 3.9 to 6.1 mM and can rise to 7.9 mM, 2 h after a meal in healthy people. Diabetic patients have plasma glucose levels that exceed 7.0 mM on an empty stomach or 11.1 mM on a 2h post-prandial test [25]. The key to effective diabetes management is the timely monitoring of varying blood glucose levels. However, the traditional finger-pricking blood test is impractical for frequent use due to poor patient compliance. An alternative is the painless and bloodless microneedle technique. Glucose concentrations ranging from 0 to 12 mM were measured using MNAs. Figure 5b shows representative images of the color change of the MNA sensor in the detection of different glucose with different concentrations and the relevant changes in the RGB percent (red) are depicted in Figure 5a. 

### 2.5. Cytotoxicity

Materials similar to those studied here have been used in MNAs for drug delivery applications and may be considered non-cytotoxic [46,52,53,54]. Although these materials are generally considered safe, their cytotoxicity at applied concentrations must be assessed to ensure their safety in medical research and clinical applications. The suggested formulation (50% PEGDA) was evaluated for this purpose using a cell viability assay experiment. The results of the tests revealed that the tested material could be used to create MNAs for detection applications. As shown in Figure 5c, the decrease in cell viability after seven days is not statistically significant compared to the first day of incubation.

## 3. Conclusions

We show that a 3D-printed PEGDA microneedle can be used to extract skin ISF and detect biomarkers. The influence of PEGDA concentration and needle diameter on mechanical strength and extraction properties was investigated, and optimal MNA specifications were proposed. The MNA includes a multiplexed sensor for insitu colorimetric detection of multiple biomarkers in extracted interstitial fluid (specifically pH and glucose). The microneedle patch is simple to apply and can be detected using the naked eye. Smartphones installed with specially developed apps can be used for quantitative RGB analysis. It is also possible that other metabolites (such as cholesterol and lactate) could be tested in the same way. The device developed has the potential to be used in medical practice for chronic diseases.

Based on the outcomes, a microneedle with a hexagonal cross-section shape and 50% concentration of PEGDA is suggested for detection as it needs a lower penetration force and higher absorption capacity than the other studied possible MNAs in this research.

## 4. Materials and Methods

### 4.1. Materials

PEGDA (Mw 700 Da), LAP, tartrazine, bromocresol green, α-naphtholphthalein powder, glucose, horseradish peroxidase, glucose oxidase, potassium iodide, trehalose, sodium citrate, and Dowex 1 × 4 chloride foam beads were purchased from Sigma-Aldrich (St. Louis, MO, USA). Fetal bovine serum (FBS), phosphate-buffered saline (PBS), and Dulbecco’s modified Eagle’s medium (DMEM) were purchased from Gibco (Grand Island, NE, USA). Presto blue (PB) reagent was purchased from Invitrogen (A13261, Waltham, MA, USA). Sodium citrate was purchased from VWR, Radnor, PA, USA.

### 4.2. Study Design

This study aims to investigate the feasibility of using PEGDA hydrogel MNAs for ISF detection applications and to find optimum specifications for it. In this regard, the fabrication of MNAs using the 3D printing process is assessed, and then the capability of MNAs with different specifications for liquid extraction and skin penetration, and colorimetric sensing is evaluated. Based on the outcomes, the MNA with the best specifications is suggested. 

### 4.3. Fabrication of MNAs

For preparing the PEGDA-based bioink, 0.3 wt% of LAP and 2.5 mM of tartrazine (concentrations in the final bioink) were dissolved in distilled water and then the required amount of PEGDA was added to the solution. The bioink was shaken before printing. The Lumen-X DLP bioprinter manufactured by Cellink Co. (Gothenburg, Sweden) was used to print MNAs. After printing, the MNAs were rinsed with distilled water and dried in air at room temperature. 

### 4.4. Synthesis of pH-Sensitive Beads

α-naphtholphthalein powder was dissolved in 100% ethanol to produce a stock solution at 100 mg/mL in a 15 mL conical. 1M NaOH was added in a dropwise manner to get all dye into the solution if necessary. Next, 400 g of Dowex resin was suspended in 400 mL of distilled water in a 500 mL beaker. While constantly stirring the Dowex solution with a glass stir rod, the dye stock solution was added in a dropwise manner using a pipette to a final concentration of 2 mg dye/1 g of beads. The resin was allowed to sit undisturbed until it settled to the bottom of the beaker (approximately 10–15 min). As much water as possible was then removed from the beaker using a 26 mL syringe. Beads were washed 3 times in distilled water by repeating this process. After the final wash, the beads were transferred to a size 25 mesh fabric sieve placed on a funnel positioned over the waste beaker. They were allowed to drain for approximately 1–2 min. The beads and mesh were then moved onto a few paper towels to help them drain further. After drying, the beads were stored in a sealed jar at room temperature.

### 4.5. Synthesis of Glucose-Sensitive Beads

A glucose-sensitive solution was prepared by dissolving 6 mg of glucose oxidase, 0.6 mg of horseradish peroxidase, 498 mg of potassium iodide, and 513 mg of trehalose in 5 mL of sodium citrate buffer (pH 6). Next, 2000 mg of Dowex 1 × 4 chloride foam beads were washed 3 times with distilled water and once with anhydrous ethanol. Following, ethanol was aspirated, it was replaced with the glucose-sensitive solution, and incubated at 4 °C overnight to allow the dye to conjugate to the beads. The solution was aspirated, and the beads were washed with distilled water 4 times, leaving the glucose-sensitive beads.

### 4.6. Fabrication of Colorimetric Sensor

Dye-loaded beads were formed in tablet shape using a 3D printed die. A separator was used to make tablets with two beads (Figure S1).

### 4.7. Swelling Experiment

PBS solution was used to investigate the ability of MNAs to extract ISF. MNAs with different PEGDA concentrations were placed in PBS solution for 10 min and the increase in the diameter of needles was measured as an index for liquid absorption and swelling. 

### 4.8. Penetration Experiment

The mechanical strength testing was performed using a custom-made compression test device. MNAs with different needle diameters and different cross-section shapes penetrated pig skin and the changes in penetration force against displacement were plotted. 

### 4.9. Sensing Experiment

A polydimethylsiloxane (PDMS) mold with cavities (Figure 1d) was used to investigate the sensing of MNAs. pH solution (NaOH or bromocresol green) and glucose were filled in the cavities for the pH and glucose sensing experiment, respectively. MNAs were placed in the cavities so that the needles were put into the solution. After absorption of the solution by MNA and the reaching of the solution to the sensor, the image of color change was captured and analyzed by Image J software, and the RGBs were plotted. 

### 4.10. Cell Viability Assay

In 96-well plates with 10,000 cells per well, the 3D-printed hydrogel-based MNAs were cultured in 0.2 mL of media for 24 h to determine their cell viability. By monitoring the metabolic activity of the live cells, the Presto blue (PB) assay was used as a standard procedure for quickly assessing the viability and proliferation of a variety of cell types. To evaluate the experimental samples’ cell viability, an indirect extraction method was used. For 1, 3, 5, and 7 days, 3D-printed MNAs were incubated in triplicate in 1 mL of DMEM containing 10% *v*/*v* of FBS at 37 °C, in 5% CO_2_, and in fully humidified air. At each interval, 500 L of the extraction medium was collected for the PB assay and replaced with a fresh DMEM + FBS solution. The cells were then given 200 µL of each periodically collected extract, and they were allowed to incubate for 24 h. Next, a PB cell viability reagent was used to determine the metabolic activity of the cells. A 100 µL volume of 10% PB reagent was prepared in DMEM solution and added to each well. The plate was then incubated for 15 min. The fluorescent intensity of the incubated cells was measured using 560 and 590 nm as the excitation and emission wavelengths. The following equation was then used to determine the samples’ viability:

Cell Viability: (Average fluorescent density of the samples/Average fluorescent density of the control) ∗ 100.

### 4.11. Image Processing

Optical images of the MNAs after being applied were analyzed by the RGB Histogram plugin in ImageJ. The statistical analysis was performed using GraphPad Prism 9.0 software.

### 4.12. Statistical Analysis

Data are presented with average values and standard deviation (SD) for the three experiments. One-way analysis of variance (ANOVA test) was employed for statistical analyses to assess multiple assessments. A *p*-value below 0.05 was considered statistically significant. 

## Figures and Tables

**Table 1:** Different formulations to study the swelling properties of MNAs.

Ingredients	Formulations				
	15%	32.50%	50%	67.50%	85%
PEGDA (vol%)	15	32.5	50	67.5	85
LAP (wt%)	0.3	0.3	0.3	0.3	0.3
Tartrazine (mM)	2.5	2.5	2.5	2.5	2.5
Distilled water (vol%)	85	67.5	50	32.5	15

**Table 2:** Contact perimeter and number of vertexes for MNAs with different cross-section shapes.

Cross-Section Shape	Cross-Section Perimeter (mm^2^)	Number of Vertices
Round	1.885	0
Triangle	1.559	3
Square	1.697	4
Hexagon	1.8	6

## Data Availability

The data presented in this study are available on request from the corresponding author. The data are not publicly available due to privacy restrictions.

## Supplementary Materials

The following supporting information can be downloaded at: https://www.mdpi.com/article/10.3390/polym15061389/s1. Figure S1: A 3D printed device set for making the multiplexed sensor.

## Abbreviations

3D	3-dimensional	MNA	Microneedle array
CAD	Computer-aided design	PB	Presto Blue
DLP	Digital light processing	PBS	Phosphate-buffered saline
DMD	Digital micromirror device	PDMS	Polydimethylsiloxane
DMEM	Dulbecco’s modified eagle medium	PEGDA	Poly(ethylene glycol) diacrylate
FBS	Fetal bovine serum	RGB	Red, green, blue
ISF	Interstitial fluid	SC	Stratum corneum
LAP	Lithium phenyl-2,4,6-trimethylbenzoylphosphinate	SD	Standard deviation
MN	Microneedle	SEM	Scanning electron microscopy
