# Supplementary material for: 3D Printed Hydrogel Microneedle Arrays for Interstitial Fluid Biomarker Extraction and Colorimetric Detection
Source: Polymers (Basel). 2023 Mar 10;15(6):1389. doi: 10.3390/polym15061389 (PMC10054006; doi:10.3390/polym15061389)
Supplement: Supplementary file 1 [file polymers-15-01389-s001.zip › polymers-2223714-supplementary.pdf]

polymers

MDPI

Article

3D Printed Hydrogel Microneedle Arrays for Interstitial Fluid Biomarker Extraction and Colorimetric Detection

Mahmood Razzaghi [1], Amir Seyfoori [1], Erik Pagan [1], Esfandyar Askari [1], Alireza Hassani Najafabadi [2] and Mohsen Akbari [1,2,3,] *

[1] Laboratory for Innovations in Microengineering (LiME), Department of Mechanical Engineering, University of Victoria, Victoria, BC V8P 5C2 Canada
[2] Terasaki Institute for Biomedical Innovations, Los Angeles, CA 90050, USA
[3] Biotechnology Center, Silesian University of Technology, Akademicka 2A, 44-100 Gliwice, Poland
* Correspondence: makbari@uvic.ca

Support information

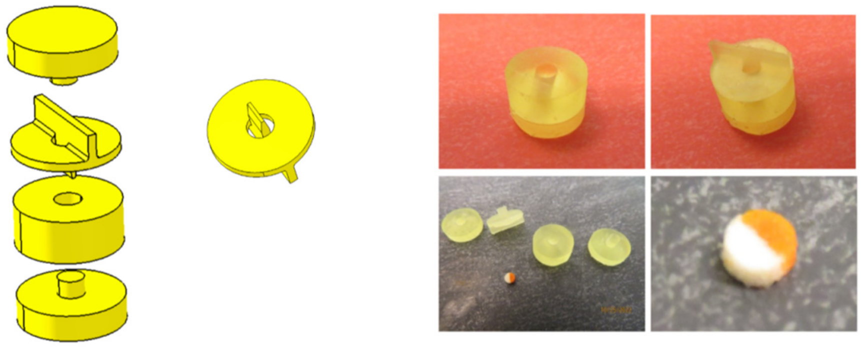

Figure S1. A 3D printed device set for making the multiplexed sensor.
